# Peer Review for “Administration Technique of Intranasal Corticosteroid Sprays Among Nepali Pharmacists: Cross-Sectional Study”

**DOI:** 10.2196/91439

**Published:** 2026-01-29

**Authors:** Sunny Chi Lik Au

**Affiliations:** 1 Tung Wah Eastern Hospital, So Kon Po, China (Hong Kong)

**Keywords:** intranasal corticosteroid spray, allergic rhinitis, device use technique, pharmacist, patient counselling, continuing pharmacy education

*This is a peer-review report for “Administration Technique of Intranasal Corticosteroid Sprays Among Nepali Pharmacists: Cross-Sectional Study.”*

## Round 1 Review

### General Comments

This paper [1] addresses an important gap by evaluating pharmacists’ proficiency in demonstrating intranasal corticosteroid technique, using a standardized 12-step checklist with 5 critical steps. The sample size (n=365) is reasonable for a local study, and the use of multivariate logistic regression and Chi-square automatic interaction detection decision tree analysis adds analytical depth. The findings highlight systemic issues, such as inadequate training and curriculum gaps, which could inform policy changes to improve allergic rhinitis management and reduce adverse effects like epistaxis.

### Specific Comments

#### Major Comments

1. Simple random sampling was used for pharmacies, but details on how wards were selected or how pharmacists within pharmacies were approached are vague. Please supplement and elaborate on further details of the randomization. More information on such would help lower the selection bias (eg, busier or more accessible pharmacies might be overrepresented).
2. The questionnaire’s validity is only face-validated by experts, with no content or construct validity testing mentioned. Reliability was assessed via Cronbach alpha (0.758) on a small pilot (n=15), which is acceptable but not robust. The cutoff for “adequate” proficiency (>6/12 marks) is based on the median score and expert opinion, which feels arbitrary and not clinically validated. Why not base it on critical steps alone, given their emphasis on efficacy and safety? Only 6% performed all 5 critical steps correctly, yet 47% were deemed “adequate” overall. This discrepancy suggests the threshold may be too lenient, masking true incompetence in high-impact areas like directing the nozzle away from the septum (to prevent epistaxis) or exhaling through the mouth (to optimize deposition). Please address these in the Discussion section.
3. Self-reported variables (eg, counseling frequency, use of materials) are prone to recall or social desirability bias, especially in an in-person interview setting. Please supplement these in the Discussion section.
4. The multivariate binary logistic regression identifies associations (eg, male gender, older age, higher qualifications linked to better proficiency), but potential confounders like pharmacy type (independent vs chain) or workload details are not controlled for. Odds ratios are extreme in places (eg, BPharm holders 97% less likely to perform inadequately, or frequent counselors 11 times more proficient), which may stem from small subgroups or multicollinearity.
5. Gender differences (males ~2 times more proficient) were found but underlying factors were not explored (eg, access to workshops, cultural biases). Please elaborate more or address the potential underlying factors in the Discussion section.
6. “Educational materials” are linked to better proficiency, but what constitutes these (eg, leaflets, videos)? Please specify for readers to enhance the proficiency on applying the study’s results.
7. Reference 16 has the wrong format for the volume, issue, and page numbers:Al-Taie A. A Systematic Review for Improper Application of Nasal Spray in Allergic Rhinitis: A Proposed Role of Community Pharmacist for Patient Education and Counseling in Practical Setting. Asia Pacific Allergy. 2025:10-5415.The full information from PubMed is as below:Al-Taie A. A systematic review for improper application of nasal spray in allergic rhinitis: A proposed role of community pharmacist for patient education and counseling in practical setting. Asia Pac Allergy. 2025 Mar;15(1):29-35. doi: 10.5415/apallergy.0000000000000173. Epub 2025 Jan 13. PMID: 40051424; PMCID: PMC11882221.Therefore, “2025:10-5415” should be “2025 Mar;15(1):29-35.”Please revise the whole reference list to see if any other typos exist.
